# An effective method to resolve ambiguous bisulfite-treated reads

**DOI:** 10.1186/s12859-021-04204-6

**Published:** 2021-05-27

**Authors:** Mengya Liu, Yun Xu

**Affiliations:** 1 School of Computer Science, University of Science and Technology of China, Hefei, 230027 Anhui China; 2 Key Laboratory on High Performance Computing of Anhui Province, Hefei, China

**Keywords:** DNA, Methylation, Bisulfite, Multireads

## Abstract

**Background:**

The combination of the bisulfite treatment and the next-generation sequencing is an important method for methylation analysis, and aligning the bisulfite-treated reads (BS-reads) is the critical step for the downstream applications. As bisulfite treatment reduces the complexity of the sequences, a large portion of BS-reads might be aligned to multiple locations of the reference genome ambiguously, called multireads. These multireads cannot be employed in the downstream applications since they are likely to introduce artifacts. To identify the best mapping location of each multiread, existing Bayesian-based methods calculate the probability of the read at each position by considering how does it overlap with unique mapped reads. However, $\sim 25$% of multireads are not overlapped with any unique reads, which are unresolvable for existing method.

**Results:**

Here we propose a novel method (EM-MUL) that not only rescues multireads overlapped with unique reads, but also uses the overall coverage and accurate base-level alignment to resolve multireads that cannot be handled by current methods. We benchmark our method on both simulated datasets and real datasets. Experimental results show that it is able to align more than 80% of multireads to the best mapping position with very high accuracy.

**Conclusions:**

EM-MUL is an effective method designed to accurately determine the best mapping position of multireads in BS-reads. For the downstream applications, it is useful to improve the methylation resolution on the repetitive regions of genome. EM-MUL is free available at https://github.com/lmylynn/EM-MUL.

## Background

The vital epigenetic mechanisms include DNA methylation, histone modification, genetic imprinting, etc. The most widely studied are PTM [1–3] and DNA methylation [4–6]. DNA methylation is an important component of epigenetic that affects the expression of genes without changing the gene sequence, opening up new ways for cancer diagnosis and treatment [7]. Bisulfite sequencing (BS-seq), which combines the bisulfite treatment with the next generation sequencing (NGS), is the gold standard for methylation analysis [8]. It coverts unmethylated cytosine (C) to thymine (T), while keeps methylated C unchanged [9]. As a result, the alignment of BS reads to the reference genome should be performed asymmetrically. That is, each T in BS reads might be aligned to T or C in the reference genome [10], but not vice versa. Owing to the reduced complexity of the BS reads and the asymmetric alignment challenge, the BS reads are more likely to be ambiguously aligned to the reference genome at multiple locations, called multireads [9]. In contrast, the reads that are uniquely aligned to its best position are named unique reads. If the alignments of multireads are used in the downstream analysis, artifacts would be introduced in the DNA methylation results. Therefore, these multireads are discarded in practice, which leads to the waste of sequencing depth and makes the methylation status of repetitive regions unresolvable [11].

There are several statistical Bayesian-based attempts that are designed to identify the best mapping position of each multiread [12–15]. Most of them make use of known information, such as the mapping quality of multiread’s aligned positions, to select the most likely one. These methods present high accuracy only if multireads are overlapped with unique reads. To further improve the accuracy, the alignment coverage of unique reads onto the reference genome are also used [16]. Besides, [17] modified the scoring matrix to classify the mismatches and the indels into different types using the base abundances of 3’ end.

Here we propose EM-MUL, a novel method combining all ideas above. For the multireads overlapped with unique reads, we use a comprehensive scoring strategy to jointly consider the similarity among sequences, bisulfite treatment, methylation region information, as well as probabilities of sequencing errors. For the remaining multireads without any overlaps to unique reads, our method assigns the locations of these multireads to achieve uniform coverage of the genome wide. This paper is organized as follows. In the results and discussion section, we briefly introduce the real and simulated data sets of bisulfite sequencing in our experiments in order to compare EM-MUL with existing methods. Moreover, we give the alignment results of EM-MUL on multiple BS-reads datasets with different read lengths, coverage depths and sequencing error rates. In next section, we summarize the experimental results. In final section, our method are given in detail.

## Results and discussion

### Data generation and analysis

#### Real data

The real data sets we use are GSM1163695, GSM4558210 and GSM4558212, which are mentioned in article [14, 18, 19]. The first data set is the bisulfite sequencing data of the human frontal cortex, which includes ten parts, each with about 100 million single reads. The length of reads is 101bp. Other data sets are the bisulfite sequencing data of mouse embryos and the length of reads is 100bp. Randomly select 1% from the unique reads and shorted the length (i.e., 25bp shorter than original reads), so that part of the shorted reads is aligned to multiple positions, and the positions of the unique reads are used as the standard to verify accuracy.

#### Simulated data

We use Mason2 [20] and Sherman [21] to simulate BS-reads, which have been used many times in previous studies [22–24]. Sherman can better simulate the real data set. However, due to the structural variation, insertion and deletion in the simulated BS-reads, it is not possible to output accurate alignment positions. Mason2 can simulate SNP sites, generate sequencing errors, and also output alignment positions. It is helpful for the verification of our results. The reference genomes we use are the human genome (hg38), the mouse genome (mm38) and the Arabidopsis (tair10). The default parameters are as follows. The length is 100bp, the SNP rate is 0.001, the methylation conversion rate in CG is 70% and in CH is 0.5%. The average coverage depth is 20X and the sequence error rate is 0.01. All our experiments are run on an Intel(R) Xeon(R) Gold 5120 GPU @ 2.20GHz machine with 28 cores and 512GB of memory.

### Evaluation measures

Here, our method is compared with BAM [14], random selection [16] and other methods [25–29]. The evaluation criterions are accuracy, recall and ${{F}_{1}}$ value. The accuracy (*p*) refers to the correct proportion of multireads we found. The recall (*r*) is the proportion of multireads that finds the unique position. The ${{F}_{1}}$ (Eq.1) value comprehensively considers both the accuracy and the recall. It can be used to measure the overall quality of the method.

1 $$\begin{aligned} {{F}_{1}}=\frac{2\times p\times r}{p+r}. \end{aligned}$$

In addition, we divide multireads into several groups according to the numbers of alignment positions to explore the effect of different methods on each sub-dataset. The evaluation criterion is the *PerRight*(*i*), which means the proportion of multireads correctly aligned by different methods. The *PerRight*(*i*) is calculated using Eqs. 2 and 3, where *i* indicates that this part of multireads is aligned to *i* positions of the reference genome. ${{n}_{random}}$ is the number of multireads aligned to the correct position by a random selection method, and ${{n}_{our}}$ is the number of correctly aligned multireads processed by our method. $PerRight{{(i)}_{random}}$ and $PerRight{{(i)}_{our}}$ are the number of multireads correctly handled by the two methods, respectively.

2 $$\begin{aligned} PerRight{{(i)}_{random}}=\frac{{{n}_{random}}}{{{n}_{random}}+{{n}_{our}}} \end{aligned}$$

3 $$\begin{aligned} PerRight{{(i)}_{our}}=\frac{{{n}_{our}}}{{{n}_{random}}+{{n}_{our}}} \end{aligned}$$

### Compared with other methods

#### Results on real data

**Fig. 1:**
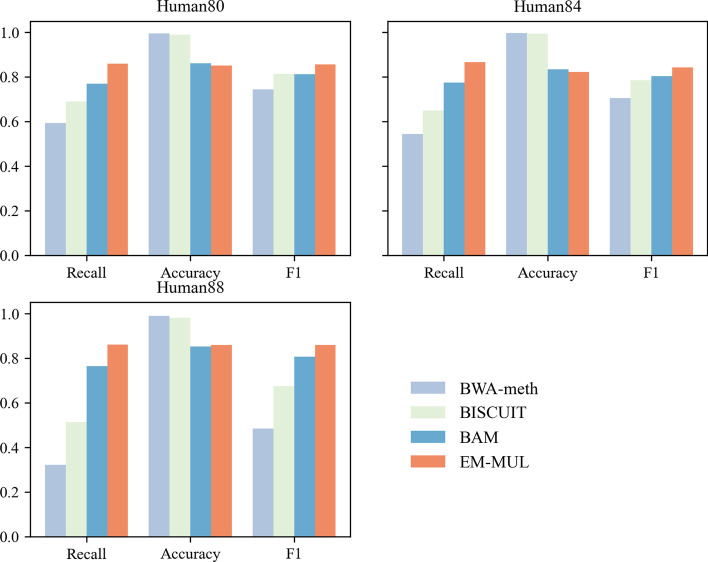
Comparison between BWA-meth, BISCUIT, BAM and EM-MUL on real human data sets. We choose three real human data sets SRR901380, SRR901384 and SRR901388. The horizontal ordinate of each picture represents recall, accuracy and F1, and the ordinate represents the value. We display the results of BWA-meth, BISCUIT, BAM and EM-MUL in turn

As shown in Fig. 1, for real human data sets, the experiments show that the accuracies of BWA-meth and BISCUIT are higher, but the recalls are lower. Between them, both the recall and F1 value of BISCUIT are higher than BWA-meth, and the accuracy of BWA-meth is higher than BISCUIT. The recall of EM-MUL is about 85%, which is $\sim 9$% higher than BAM. The accuracy between our method and BAM is less than 1%. Compared with other methods, EM-MUL can obtain a higher F1 value and align more multireads to the right position. For all tools, the alignment results which MAPQ is 0 are excluded as in [14].

**Fig. 2:**
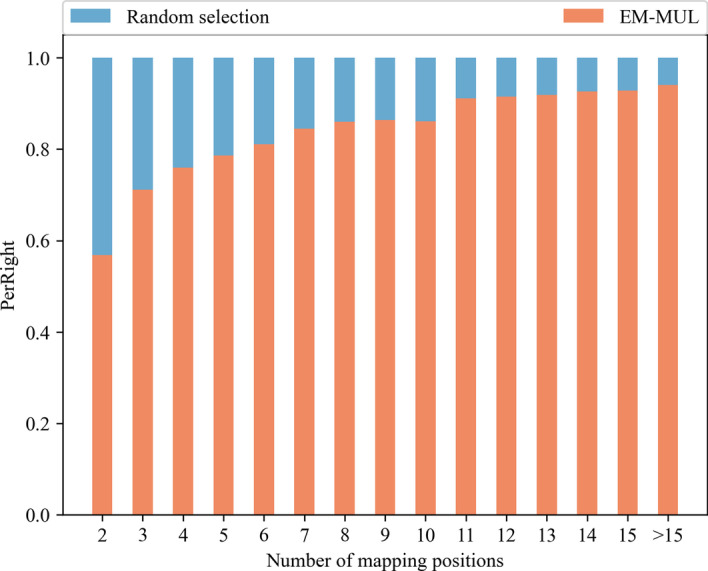
Comparison with random selection method on the real human data set. The data set we choose is SRR901388. After using Bismark to align all BS-reads to the reference genome, all possible alignment positions of multireads are obtained. We divided multireads into several groups according to the numbers of alignment positions to explore the effect of different methods on each dataset. The horizontal ordinate is the number of multiread’s mapping positions. The ordinate in the figure means the proportion of multireads correctly aligned by different methods

The random selection method is to randomly select one of the positions when there are multiple positions with the same similarity score. As shown in the first column of Fig. 2, the number of multireads aligned to the correct positions is not much different between the two methods. When the number of positions is 11, the ratio of the numbers of reads aligned to the correct position after using two methods is about 1:9. The more alignment positions of multireads, the more difficult it is for the random selection method to obtain the correct alignment positions. The advantages of the EM-MUL method have gradually become prominent.

**Fig. 3:**
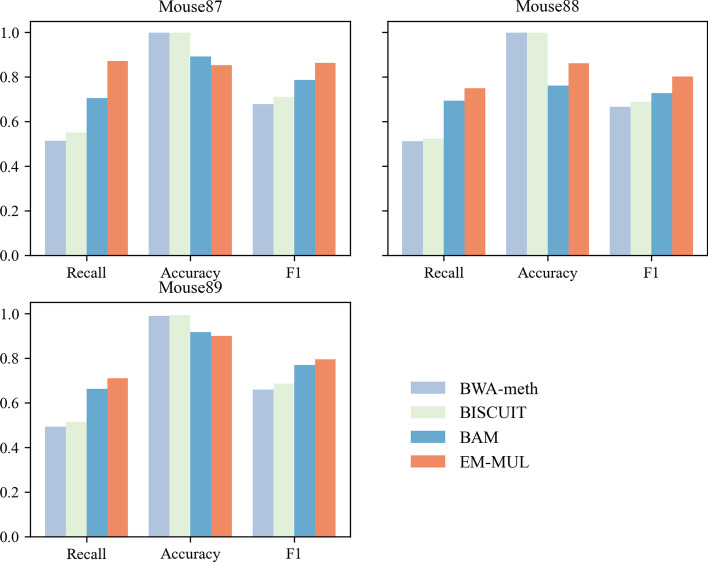
Comparison between BWA-meth, BISCUIT, BAM and EM-MUL on real mouse data sets. We choose three real mouse data sets SRR11806587, SRR11806588 and SRR11806589. The horizontal ordinate represents recall, accuracy and F1, and the ordinate represents the value

The results of the mouse data sets show that BWA-meth and BISCUIT can get high accuracy (see Fig. 3). The recalls of BAM and EM-MUL are higher than the other methods, and EM-MUL can obtain the highest F1 value. The results of EM-MUL on mouse datasets show no obvious effect on human datasets. This may be due to the higher frequency of T and C in the mouse reference genome, which is difficult to determine the unique position.

#### Results on simulated data

**Fig. 4:**
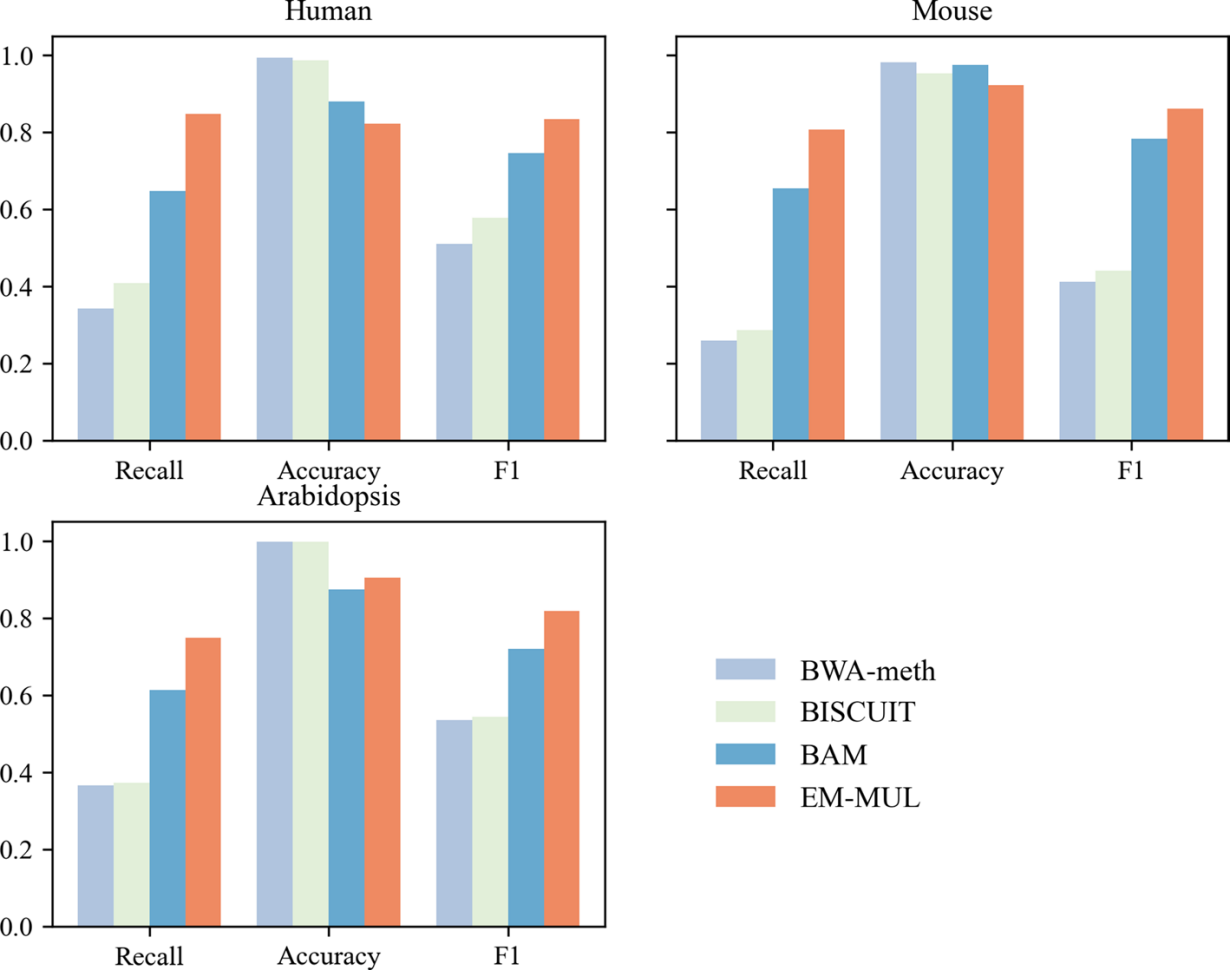
Comparison between BWA-meth, BISCUIT, BAM and EM-MUL on simulated data sets. The human, mouse and Arabidopsis simulated data sets are used separately, which are generated using the default parameters described above. The evaluation index is recall, accuracy and the F1 value

As shown in Fig. 4, for human data set, our method could assign 84% of multireads to the best locations with an accuracy rate of 82.3%. The BAM method could assign $\sim 64$% of multireads to unique locations with the accuracy of 88%. Our method align more $\sim 20$% multireads to a unique position, which only slightly affected the accuracy. BWA-meth and BISCUIT can align $\sim 30$% of the multireads to unique positions with the accuracy of $\sim 99$%. For mouse data set, multireads of 80% are aligned to unique locations. The accuracy is higher than 90%. For Arabidopsis data set, our method aligns $\sim 75$% of multireads to unique locations with higher accuracy. The ${{F}_{1}}$ value of our method is higher than other methods in all three data sets, which is the best for mouse data set, next to human data set and last to Arabidopsis data set. It is because that the multiple alignment positions of multireads in the Arabidopsis data set are too similar. Our method can perform better on the simulated data sets. The reason is that our method can be more accurate when the sequence length is longer and the number of overlapping unique reads is larger.

**Fig. 5:**
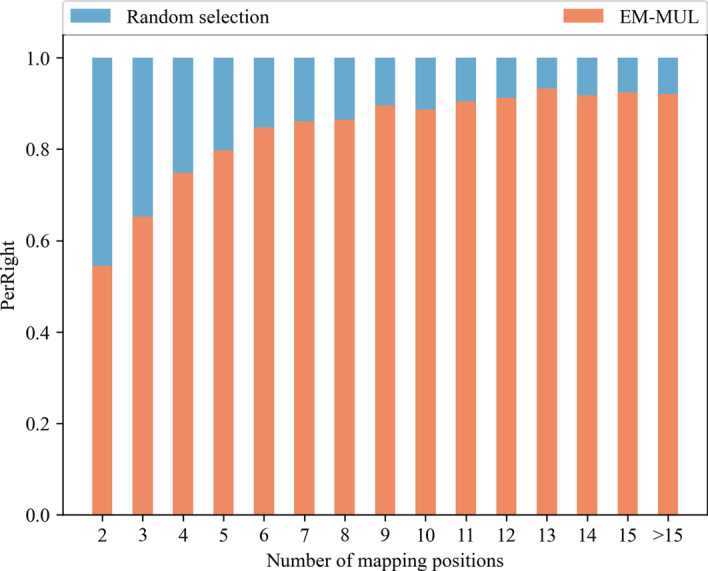
Comparison with random selection method on simulated human data sets. The data set we use is simulated human data sets with a length of 100bp. The horizontal ordinate is the number of multiread’s mapping positions. The ordinate is the proportion of multireads correctly aligned by different methods

Figure 5 compares the EM-MUL method with the random selection method, based on the correct number of multireads. It can be seen that in the data set with the alignment position $i=2$, we correctly find 13% of multireads more than the randomly selected method. When $i=15$, the number of correctly found multireads is 85.6% of multireads more than randomly selected. As the alignment position *i* grows, the proportion of multireads our method finds correctly increases.

We also compare our method with other aligners, such as BWA-meth [25], BatMeth2 [26], GEM3 [27], BISCUIT [28] and GSNAP [29]. The parameters used are listed in Table 1. As shown in Table 2, our method have the highest recall and F1 value compared with other methods. In Table 2, the bold fonts represent the best results in each evaluation criterion. The experiment here considers all BS-reads, which is different from the previous experiments for the multireads. At the same time, the determination of the unique position is very beneficial to the analysis of methylation information, which will be confirmed in the following experiments.

**Table 1:** Different parameters of methods compared

Software	Version	Arguments
BWA-meth	0.2.2	-Threads 16
BatMeth2	1.0	-p 6 -n 2
GEM3	3.6.0	default
BISCUIT	0.3.16	-t 6
GSNAP	2015-09-21	-A sam -t 6
EM-MUL	–	Default

**Table 2:** Effect of different read lengths on EM-MUL method using human, mouse and Arabidopsis simulated data sets

Tools	Human 76bp			Human 100bp		
	Recall (%)	Accuracy (%)	F1 (%)	Recall (%)	Accuracy (%)	F1 (%)
BWA-meth	87.42	**99.70**	93.15	93.11	**99.73**	96.31
BatMeth2	87.32	98.57	92.61	92.79	98.89	95.74
GEM3	89.01	98.26	93.40	94.31	98.87	96.54
BISCUIT	88.81	99.57	93.88	94.10	99.67	96.8
GSNAP	90.45	97.53	93.86	94.35	98.22	96.25
EM-MUL	**95.16**	97.26	**96.20**	**97.74**	97.88	**97.81**

**Fig. 6:**
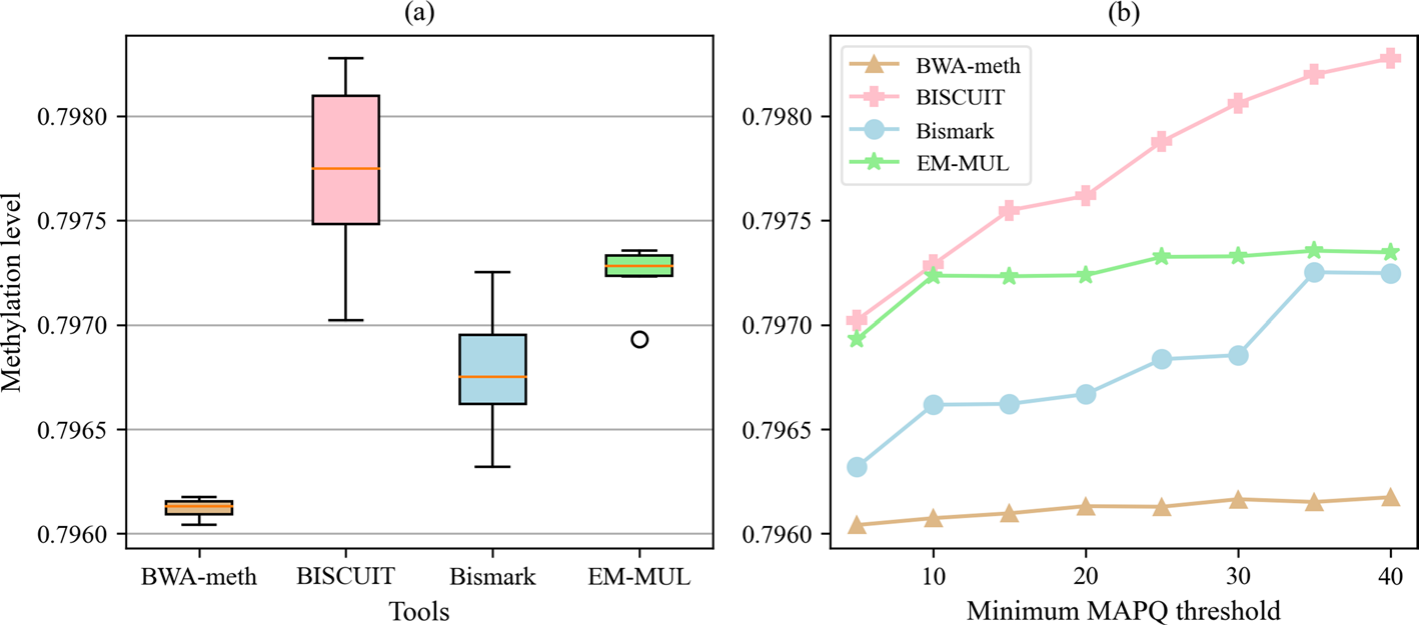
The influence on the methylation level at CpGs for different methods and different MAPQ thresholds. **a** represents the changes in the methylation level at CpGs after treatment with different tools and **b** lists the specific values. The horizontal ordinate is the different minimum MAPQ threshold selected during processing, and the ordinate is different methylation levels at CpGs

We use MethylDackel to infer the methylation level after using BWA-meth, BISCUIT, Bismark, EM-MUL and BAM. And the result is shown in Fig. 6. The data set we use is the human data with the length of 100bp, and the methylation rate at CpGs is 80%, which represents the level of methylated cytosines in CG-context. In other word, it means that 20% of CG-cytosines will be converted into thymines. It can be seen from Fig. 6a that the result of BISCUIT is closest to the true methylation level at CpGs, followed by EM-MUL, Bismark and BWA-meth. But the fluctuation range of EM-MUL is smaller than BISCUIT. It can be seen from Fig. 6b that when the threshold of minimum MAPQ increases, the error between the methylation level and the true value is also smaller. When filtering with different MAPQ thresholds, the methylation level of BAM is between 74.1% and 79.6%. Since the methylation levels of different tools are close, the results of BAM are not shown in Fig. 6 for better discrimination. Compared with Bismark, our method is closer to the true methylation level.

### Effect of different parameters for our method on simulated data

#### Read length

Table 3 shows the effect of read length on the results. The BS-reads is simulated with lengths of 76bp, 100bp and 150bp, respectively. As we can see, our method can achieve better results at different read lengths. For human data sets, different read lengths have little effect on the results, with recall ranging from 85 to 87% and accuracy rate ranging from 76 to 80%. For the mouse data set and the Arabidopsis data, as the length of the simulated data sequence increases, the accuracy increases slightly. For the Arabidopsis data set, there is also a small increase in recall rate.

**Table 3:** Effect of different read lengths on EM-MUL method using human, mouse and Arabidopsis simulated data sets

Read length	Human		Mouse		Arabidopsis	
	Recall (%)	Accuracy (%)	Recall (%)	Accuracy (%)	Recall (%)	Accuracy (%)
70bp	86.00	76.90	80.63	88.80	73.92	86.10
100bp	85.42	79.62	79.89	89.81	75.05	87.68
150bp	86.39	77.80	77.28	90.36	75.05	88.80

#### Methylation rate at CpGs

We generate BS-reads with the methylation rate at CpGs of 70%, 80%, 90% respectively and other parameters remain unchanged. It means the value of methylated cytosines in CG-context. When the value is 80%, it means 20% of CG-cytosines will be converted into thymines. We can see that the methylation rate at CpGs has little effect on our method (see Table 4). For the human data sets, the recall rate is about 85%, and the accuracy is about 79%. For the mouse data sets, the recall reaches 80% , the accuracy is about 90% and different methylation values at CpGs have a slightly negative effect on the results. For the Arabidopsis genome, the recall is from 74 to 75%, and the accuracy is about 88%. Different methylation rates at CpGs have little effect on the human and the Arabidopsis data sets.

**Table 4:** Effect of different methylation rates at CpGs on EM-MUL method using human, mouse and Arabidopsis simulated data sets

CpG rate (%)	Human		Mouse		Arabidopsis	
	Recall (%)	Accuracy (%)	Recall (%)	Accuracy (%)	Recall (%)	Accuracy (%)
70	85.24	79.41	79.91	89.83	74.31	88.02
80	85.42	79.62	79.89	89.81	75.05	87.68
90	85.22	79.34	79.86	89.79	74.31	87.97

#### The average coverage depth

**Fig. 7:**
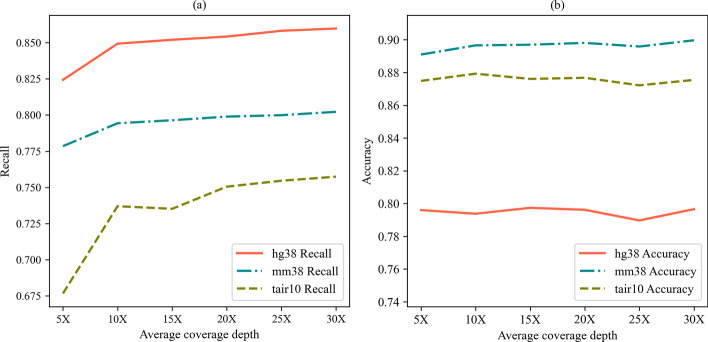
The effect of average depth of coverage. **a** represents the influence of average depth of coverage depth on recall and **b** represents the influence of average depth of coverage on accuracy

The average depth of coverage of the simulated data sets is from 5X to 30X. With the increase of the coverage depth, the recalls and accuracies on both human and mouse data sets have increased slightly, and the accuracy has increased on the Arabidopsis data set, but have little effect on the recall as shown in Fig. 7.

#### Sequencing error rate

**Fig. 8:**
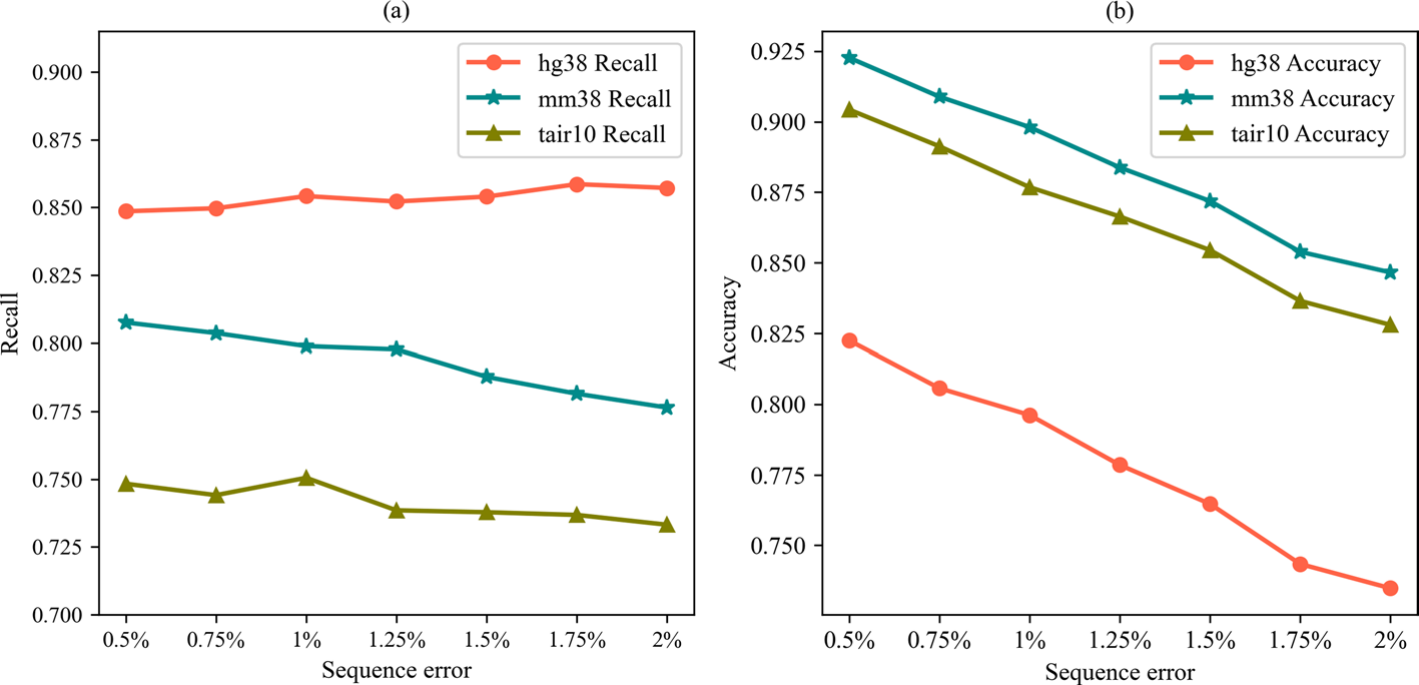
The effect of the sequence error rate. **a** represents the influence of the sequence error rate on recall and **b** represents the influence of the sequence error rate on accuracy

The data sets we use have different sequencing error rates, with values ranging from 0.5 to 2%. Figure 8 shows the impact of sequencing error rate on the experimental results. It can be seen that as the sequencing error rate increases, the accuracy on all three simulation data sets decreases.

## Conclusions

In conclusion, due to the influence of bisulfite treatment, a large part of BS-reads are mapped to multiple locations. We proposed the EM-MUL method to find the optimal alignment position of multireads. First, our method can obtain a higher F1 value on both real and simulation data sets, which means it can align more multireads to the unique position correctly. Then, the effect of different parameters on the EM-MUL method was verified. The results suggest that the read length and methylation rate at CpGs had almost no effect on the performance of our method. The average depth of coverage has a positive effect on our method, and the sequence error has a negative effect on our method. Therefore, our method is robust and performs well in different read lengths and methylation rates at CpGs. The EM-MUL method can align partial BS-reads to the repeated regions, which is beneficial to the further analysis of the repeated regions. Then, we can use the information of multireads to obtain more accurate methylation analysis results.

## Methods

**Fig. 9:**
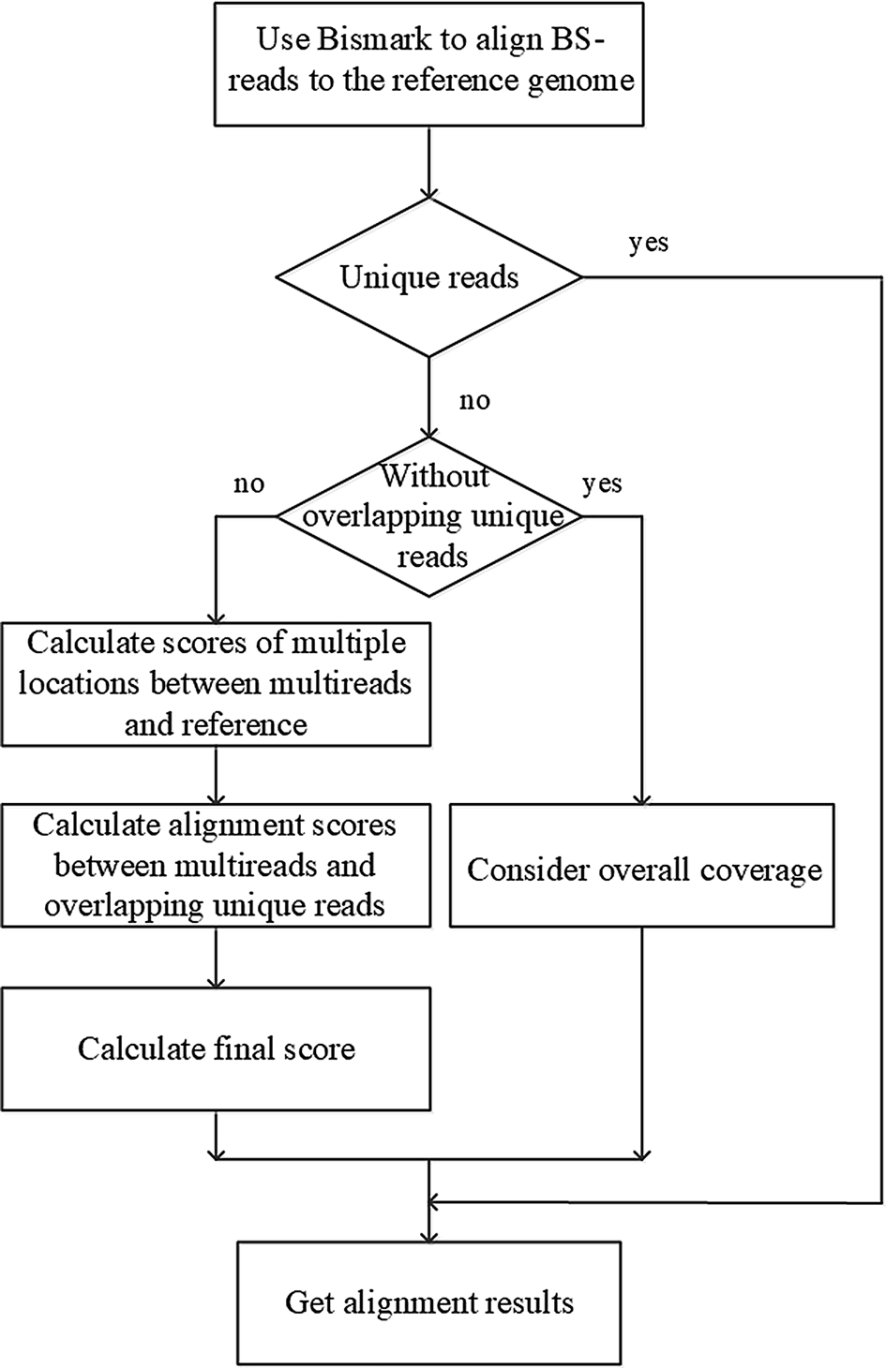
The overall process of handling multireads. First, use Bismark to align BS-reads to the reference genome to get multireads and unique reads. Then use EM-MUL to assign the most likely alignment positions of multireads

Figure 9 presents the overall workflow of EM-MUL. It employs Bismark [30] to obtain the unique reads and the multireads, and then processes the alignment results of multireads. After that, it allocates each multiread to the most likely alignment position. For multireads, we classified them into two groups according to whether they are overlapped with unique reads.

### Definitions and notations

**Fig. 10:**
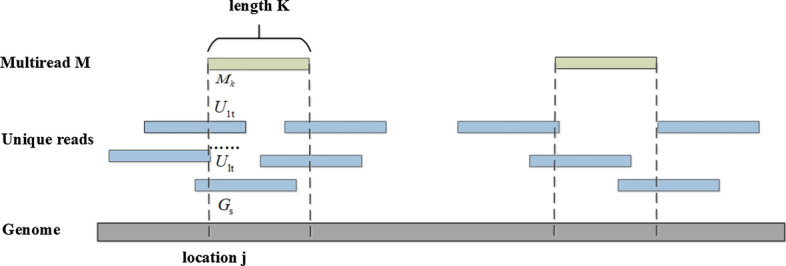
An example of the relationship between multiread *M*, overlapping unique reads and reference *G*.The multiread *M* has two alignment positions and one of them is *j*. In the left location of multiread *M*, there are *n* overlapping unique reads

Given a multiread *M* of length *K*, there are *Q* mapping locations on the reference genome *G* and one of them is the location *j* (see Fig. 10). The probability ${S}_{j}$ of *M* align to each position of the reference genome consists of two parts: the similarity ${S}_{{{M}_{k}}{{G}_{s}}}$ of *M* and reference genome, and the similarity ${S}_{{{M}_{k}}{{U}_{t}}}$ of *M* and unique reads. For the convenience of reading, all the list of symbols and notations used are provided in Table 5.

**Table 5:** Notation table

Symbol	Definition
*M*	One of the multireads
*G*	The reference genome
${M}_{k}$	The *k*-th base of the multiread *M*
${G}_{s}$	The *s*-th base of the reference genome *G*
${U}_{lt}$	The *t*-th base on the *l*-th overlapped unique reads
${S}_{j}$	Probability of *M* aligned to the *j*-th position of *G*
${{\varepsilon }_{k}}$	Probability of sequencing errors in ${M}_{k}$
${{\varepsilon }_{lt}}$	Probability of sequencing errors in ${U}_{lt}$
*loss*	A global loss function
*WinLen*	The certain length we defined
*a*[*i*]	Actual coverage of every locus in *WinLen*
$\overline{x}$	The average depth of coverage from 0 to $WinLen-1$
${S}_{{{M}_{k}}{{G}_{s}}}$	Probability of aligning ${M}_{k}$ to ${G}_{s}$, related to the base ${M}_{k}$ and ${G}_{s}$
$P({{M}_{k}},{{G}_{s}})$	Part of ${S}_{{{M}_{k}}{{G}_{s}}}$. The probability of observing ${M}_{k}$ for a given base ${G}_{s}$
$Score({{M}_{k}},{{G}_{s}})$	Part of ${S}_{{{M}_{k}}{{G}_{s}}}$. A similar score between ${M}_{k}$ and ${G}_{s}$
$S[{{G}_{s}}]$	Total similar score when the base of the reference genome is ${G}_{s}$
${S}_{{{M}_{k}}{{U}_{t}}}$	Probability of aligning ${M}_{k}$ to the *l*-th overlapping unique reads ${{U}_{t}}$
${S}_{{{M}_{k}}{{U}_{lt}}}$	Probability of aligning ${M}_{k}$ to all overlapping unique reads ${{U}_{lt}}$
$P({{M}_{k}} \rightarrow {{U}_{lt}})$	Probability of no sequencing errors occurring in both ${M}_{k}$ and ${{U}_{lt}}$
$Score({{M}_{k}},{{U}_{lt}})$	Part of $P({{M}_{k}} \rightarrow {{U}_{lt}})$. A similar score between ${M}_{k}$ and ${{U}_{lt}}$

### Multireads overlapped with unique reads

To deal with this type of multireads, here is divided into three steps. First, collect all mapping locations on the reference genome of a multiread. Second, for each position of multireads, find the unique reads that are overlapped with it. Third, give scores of the genome and unique reads on each base for any position of the multiread, add the total scores to select the best position. We will present how to score different positions in detail.

#### Calculate scores of multiple locations between multireads and reference

We choose a better scoring matrix in [17], and use more information and generate a new evaluation method. The elements in the scoring matrix are divided into insert, delete, match and mismatch, and their values are assigned according to the base abundances. Meanwhile, we also incorporate more known information, including the probability of sequencing individual mutations and the influence of bisulfite treatment. Our method calculates the probability of multiread *M* aligned to each position of the reference genome base by base. First, we adopt a scoring matrix donated as *Score* to assign different values according to the correspondence between ${M}_{k}$ and ${G}_{s}$. The matrix *Score* is shown in Table 6. The rows represent the reference genome, and the columns represent the multireads. When ${M}_{k}$ is the same as ${G}_{s}$, a positive score can be obtained as shown by the main diagonal in Table 6. In other cases, they are all negative values, except for the alignment of C on the multiread to T on the reference genome.

**Table 6:** The scoring matrix [17] for the positive strand and overlapping unique reads. Different types of match and mismatch scores are different

	A	C	G	T	N
A	6	− 18	− 18	− 18	− 25
C	− 18	6	− 18	3	− 25
G	− 18	− 18	6	− 18	− 25
T	− 18	− 18	− 18	3	− 25
N	− 25	− 25	− 25	− 25	25

Next, incorporate information such as mutation and bisulfite treatment. Let $P({{M}_{k}},{{G}_{s}})$ be the probability of observing multiread base ${M}_{k}$ for given the reference genome base ${G}_{s}$. It can be calculated by sequencing individual gene mutations and the probability of methylation in different regions. For example, when the base of the reference genome is *C*, Table 7 lists four different cases of the corresponding position between the multiread base ${M}_{k}$ and the reference genome base ${G}_{s}$. *P*(*CA*) means the probability of a *C* to *A* mutation, and *nonSNP* means the factor that is the probability of not SNP site.

**Table 7:** Four cases of the bases between the forward reference genome and multiread when the base on the reference is C

Reference base	Multiread base	Phenomenon	Calculation formula
C	A	C to A	P(CA)
C	T	Unmethylated C or C to T	P(CT)+nonSNP*P(CC)
C	C	No mutation and methylated C	NonSNP*P(CC)
C	G	C to G	P(CG)

Then, shown as Formula 4, ${S}_{{{M}_{k}}{{G}_{s}}}$ is a weighted score of aligning multiread base ${M}_{k}$ to reference genome base ${G}_{s}$. $P({{M}_{k}},{{G}_{s}})$ is the probability of observing multiread base ${M}_{k}$ given the reference genome base ${G}_{s}$. $Score({{M}_{k}},{{G}_{s}})$ is the similar score between ${M}_{k}$ and ${G}_{s}$. $S[{{G}_{s}}]$ is the total similar score when the base of the reference genome is ${{G}_{s}}$. ${S}_{{{M}_{k}}{{G}_{s}}}$ can be computed by Formula 1, which reflects the similarity of ${M}_{k}$ and ${G}_{s}$.

4 $$\begin{aligned} {{S}_{{{M}_{k}}{{G}_{s}}}}= & {} P({{M}_{k}},{{G}_{s}})\times Score({{M}_{k}},{{G}_{s}})+ \nonumber \\&(1-P({{M}_{k}},{{G}_{s}}))\times (S[{{G}_{s}}]-Score({{M}_{k}},{{G}_{s}})). \end{aligned}$$

#### Calculate alignment scores between multireads and overlapping unique reads

For the reads located at the same location, these reads are largely similar [13]. Therefore, we use the similarity between multireads and overlapping unique reads, and the locations with the highest similarity are the optimal locations. Similar to the Bayesian method, we also use the sequencing error information of unique reads overlapped with multireads to calculate the probability of multireads aligning to each position.

First, calculate the similarity between the multiread and each overlapping unique read. Using scoring matrix $Score({{M}_{k}},{{U}_{lt}})$ and $P({{M}_{k}} \rightarrow {{U}_{lt}})$ to calculate likelihood ${{S}_{{M}_{k}{U}_{t}}}$ of aligning multiread *M* to the reference genome $G[j, j+K]$. $P({{M}_{k}} \rightarrow {{U}_{lt}})$ can be computed by Formula 5.

5 $$\begin{aligned} P({{M}_{k}}\rightarrow {{U}_{lt}})=\left\{ \begin{aligned}&{{\varepsilon }_{lt}}+{{\varepsilon }_{k}}-{{\varepsilon }_{lt}}\times {{\varepsilon }_{k}}, if{{U}_{lt}}={{M}_{k}} \\&1-({{\varepsilon }_{lt}}+{{\varepsilon }_{k}}-{{\varepsilon }_{lt}}\times {{\varepsilon }_{k}}), if {{U}_{lt}}\ne {{M}_{k}}\\ \end{aligned} \right. , \end{aligned}$$

where ${{\varepsilon }_{k}}$ is the probability of sequencing errors in the base ${M}_{k}$ and ${{\varepsilon }_{lt}}$ is the probability of sequencing errors in the base ${U}_{lk}$. When ${{M}_{k}}$ and ${{U}_{lt}}$ are the same, $P({{M}_{k}}\rightarrow {U}_{lt})$ means the probability of no sequencing errors occurring in both. When ${{M}_{k}}$ and ${{U}_{lt}}$ are different, it means that at least one of the two has a sequence error. As shown in Formula 6, we define ${{S}_{{{M}_{k}}{{U}_{lt}}}}$ as follows. It is the probability of the *k*-th base of multiread *M* mapped to the corresponding position of *l*-th overlapping unique read.

6 $$\begin{aligned} {{S}_{{{M}_{k}}{{U}_{lt}}}}= & {} \sum \limits _{l=1}^{n}{P({{M}_{k}}\rightarrow {{U}_{lt}})\times Score({{M}_{k}},{{U}_{lt}})}+  \\&\sum \limits _{l=1}^{n}{(1-P({{M}_{k}}\rightarrow {{U}_{lt}}))\times (S[{{U}_{lt}}]-Score({{M}_{k}},{{U}_{lt}}))}. \end{aligned}$$

Next, calculate the similarity between *M* with anyone of the overlapping unique reads. Formula 7 can calculate the probability of the *k*-th base of multiread *M* aligned to the corresponding position related to all overlapping unique reads. *n* is the number of overlapping unique reads corresponding to the *k*-th base of multiread *M*. To reduce the impact of each unique read on the calculation result, the result of all unique reads are averaged to obtain ${S}_{{{M}_{k}}{{U}_{t}}}$, where is computed by Formula 7.

7 $$\begin{aligned} {{S}_{{{M}_{k}}{{U}_{t}}}}=\sum \limits _{l=1}^{n}{{{S}_{{{M}_{k}}{{U}_{lt}}}}/n}. \end{aligned}$$

#### Calculate final score

In this step, the scores of the first two steps are weighted to get the final alignment scores of multireads and get the determined alignment locations. First, introduce the method to obtain the final alignment score. For multiread *M*, the score of each position can get from formula 8. Through the introduction in the previous two sections, we can calculate the probability *S* of multiread *M* aligned to each position according to the reference genome and the overlapping unique reads. The reference genome and overlapping unique reads have the same weight on the final scores, both are 0.5. If there are no overlapping unique reads, then ${{S}_{{{M}_{k}}{{U}_{t}}}}$ is assigned to ${{S}_{{{M}_{k}}{{G}_{s}}}}$, and the result is calculated.

8 $$\begin{aligned} {{S}_{j}}=\sum \limits _{k=1}^{K}{\frac{{{S}_{{{M}_{k}}{{G}_{s}}}}+{{S}_{{{M}_{k}}{{U}_{t}}}}}{2}}. \end{aligned}$$

Then, get the determined alignment location of each multiread *M*. Suppose multiread *M* has *Q* mapping positions, we select the maximum value ${{S}_{max }}$ and the second largest value ${{S}_{nextmax }}$ from these *Q* positions. We think that the position with the highest score is the best alignment position, only if the condition ${{S}_{max }}-{{S}_{nextmax }}>\sigma$ is satisfied, which is a modifiable threshold to adjust the resulting error. However, due to the existence of repeated regions on the reference genome, there are still parts of multireads that cannot be allocated. Subsequent steps need to be considered in conjunction with local coverage, and the optimal alignment location of the remaining multireads is determined.

### Multireads without overlapping unique reads

This part of the processing is based on the following assumptions. After all reads are aligned to the reference genome, the overall distribution should be uniform, also known as smoothness [16]. Based on this assumption, we consider evaluating the local smoothness of different mapping positions of each multiread and choose one position of the multiread that can maintain the overall smoothness.

**Fig. 11:**
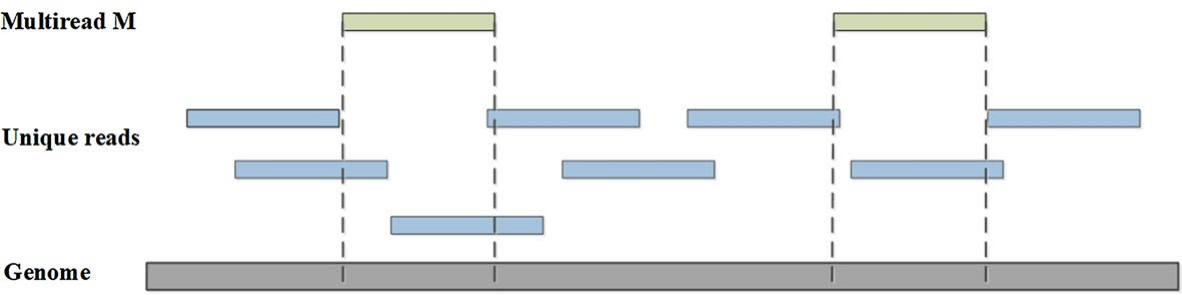
An example of getting the determined alignment location using coverage. The multiread *M* has two alignment positions. If *M* is in the left position, the average depth of coverage about it and the right will respectively more smooth than *M* in the right

First, evaluate the local smoothness of different mapping positions. For each possible alignment position of the multiread, a global loss function *loss* is calculated to represent the global non-smoothness, and the alignment position that makes the overall smoothest is selected. As Fig. 11 shows, multiread *M* has two mapping positions and unique reads about each position can be obtained to calculate local coverage. We use Formula 9 to calculate the local smoothness *loss* about a certain length of positions. *WinLen* is the certain length we defined. *a*[*i*] is the actual coverage of every locus in *WinLen*, and ${\overline{x}}$ is the average depth of coverage from 0 to $WinLen-1$.

9 $$\begin{aligned} loss=\frac{1}{WinLen}\sum \limits _{i=0}^{WinLen-1}{{{(a[i]-{\overline{x}})}^{2}}}. \end{aligned}$$

Then, choose one position of each multiread. If multiread *M* has multiple alignment positions, we will calculate between every two alignment positions and the position with a minimum value of *loss* will be selected. This step is finished when every two positions are calculated.

## Data Availability

The data sets are publicly available on NCBI databases, Accession Numbers GSM1163695, GSM4558210 and GSM4558212.
